# The Effect of Transcranial Direct Current Stimulation Combined with Visual Cueing Training on Motor Function, Balance, and Gait Ability of Patients with Parkinson’s Disease

**DOI:** 10.3390/medicina57111146

**Published:** 2021-10-22

**Authors:** Si-a Lee, Myoung-Kwon Kim

**Affiliations:** 1 Department of Rehabilitation Sciences, Graduate School, Daegu University, Jillyang, Gyeongsan, Gyeongbuk 712-714, Korea; blue1025white@naver.com; 2 Department of Physical Therapy, College of Rehabilitation Sciences, Daegu University, Jillyang, Gyeongsan, Gyeongbuk 712-714, Korea

**Keywords:** Parkinson’s disease, transcranial direct current stimulation, motor, balance, gait

## Abstract

*Background and Objectives*: The purpose of this study was to investigate the effects of transcranial direct current stimulation (tDCS) on motor function, balance and gait ability in patients with Parkinson’s disease (PD). *Materials and Methods*: For the experiment, 30 patients with PD were randomly assigned to the experimental group (*n* = 15) and the control group (*n* = 15). Visual cueing training was commonly applied to both groups, the experimental group applied tDCS simultaneously with visual training, and the control group applied sham tDCS simultaneously with visual training. All subjects were pre-tested before the first intervention, post-tested after completing all 4 weeks of intervention, and followed-up tested 2 weeks after the completing intervention. The tests used the Unified Parkinson’s Disease Rating Scale (UPDRS) for motor function assessment, Functional Gait Assessment (FGA) for balance assessment, Freezing of Gait Questionnaire (FOG-Q) and the GAITRite system for gait ability assessment. Among the data obtained through the GAITRite system, gait velocity, cadence, step time, double support time, and stride length were analyzed. *Results*: The experimental group showed a significant decrease in UPDRS and a significant increase in FGA and cadence after the intervention. In addition, UPDRS and cadence showed a significant difference in the follow-up test compared to the pre-intervention test. *Conclusions*: This study suggests that the application of tDCS to the supplementary motor area of PD patients is useful as an adjuvant therapy for rehabilitation training of PD patients.

## 1. Introduction

Parkinson’s disease (PD) is a progressive degenerative disease that is characterized clinically by tremor, rigidity, bradykinesia, and postural instability [1]. The annual incidence of PD is 4.5–19 per 100,000 population, and the prevalence is estimated to be 72–258.8 per 100,000 population [2]. PD is mainly due to the gradual decline of dopaminergic neurons in substantia nigra pars compacta, and the decline of dopaminergic neurons results in a lack of coordinated activity of the direct and indirect circuits of the basal ganglia [3]. This causes abnormal activity in the cortico-striatal-thalamic pathways of the central nervous system [4]. Motor disability occurs from the beginning of PD, and the severity of the disorder increases as the duration of the disease increases. The most common symptoms appearing from the beginning of the disease include pain, movement disorders, depression and insomnia [5]. In particular, it is estimated that more than one-third of PD patients have difficulty in starting their gait, or a freezing of gait (FOG) in which their steps suddenly stop while walking [6]. In this way, PD patients with initial hesitation and FOG have a lower anticipatory postural adjustments (APAs), which increases the risk of falls [7]. According to previous studies, more than 30% of PD patients experience fractures due to falls within 10 years after diagnosis [8]. In order to prevent secondary complications from such falls, motor function, balance, and gait ability are essential factors for PD patients.

There are several management methods available to alleviate the symptoms in PD patients. Pharmacological therapy occupies a large part of the management of PD patients, and levodopa is considered the most effective treatment for PD and is most often prescribed. However, levodopa has side effects such as abnormal involuntary movement known as dyskinesia. Drugs other than levodopa also suffer from side effects and increased resistance to drugs [9]. Surgical interventions, such as deep brain stimulation, have limited number of applicable patients, and there is a risk of serious complications and neuropsychiatric side effects [10]. Direct physical training to improve motor symptoms such as bradykinesia and FOG also plays an important part in the management of PD patients. In particular, cueing training has been frequently used recently as a motor training method specially used for PD patients.

Cueing is defined as using external stimulation such as visual or auditory stimuli to facilitate movement initiation and continuation [11]. According to previous study, it was suggested that cueing could have a significant effect on the gait performance of PD patients, i.e., cadence, stride length, speed, and postural stability [12]. The visual cueing refers to using a laser pointer or a line marked on the floor. In general, stride length markers are often used, and are arranged vertically along the walking path at regular intervals. It is assumed that these visual signals have a positive effect on walking by improving attention in the stepping process [13]. McAuley et al. showed that the use of visual cue glasses had an improvement of at least 10% in walking time [14]. However, such physical therapy or functional training methods can improve movement disorders in PD patients, but the duration of effect is limited [15]. In order to obtain the long-term effect of intervention, motor learning needs to be stored under stimulation of neuroplastic mechanisms [16].

Representative methods of non-invasive brain stimulation applied as alternatives to these are transcranial magnetic stimulation (TMS) and transcranial direct current stimulation (tDCS). TMS uses electrical stimulation induced by a magnetic field, whereas tDCS conducts direct current via a surface electrode attached to the scalp for a certain period of time [10]. In non-invasive brain stimulation, penetration is limited only to the cortical regions of the brain; thus, deep structures such as the basal ganglia cannot be directly targeted [17]. However, it has been proven through meta-analysis that stimulation of the epidural motor cortex is useful in improving the symptoms of PD patients [10].

Transcranial direct current stimulation regulates excitability in the cortex by inducing a direct current via the scalp using the anode and the cathode electrodes. The anode increases cortical excitability, and the cathode decreases cortical excitability [18]. Unlike pulse stimulation, tDCS induces changes in cortical excitability by producing sufficient action potential through continuous current. In particular, since anodal tDCS promotes activation of neurons through an increase in cortical excitability, it acts to increase the activity of the decreased motor cortex in PD patients. In addition, it has been reported that activation of the cortex by anodal tDCS induces dopamine release, thereby improving the symptoms of PD patients [19,20]. Since patients with PD develop dysfunction of the basal ganglia due to decreased dopamine secretion, applying bipolar tDCS to increase cortical excitability can compensate for the decreased pallido–halamo–cortical drive [21]. A previous study showing similar results suggested that application of high-frequency rTMS to the prefrontal cortex induces dopamine release from the tail nucleus through an increase in cortical excitability [22]. In addition, since the N-methyl-d-aspartate (NMDA) receptor is dopamine-dependent, it has been reported that the release of dopamine through the application of tDCS can induce plasticity of the NMDA receptor [23]. In a study involving PD patients, it was reported that the application of tDCS to the motor, premotor or prefrontal cortex improved gait function. Other previous study showed that the FOG was improved as a result of applying tDCS to primary motor cortex of PD patients for 4 weeks [24,25].

The supplementary motor area (SMA) is the primary output site of the basal ganglia thalamocortical pathway and plays an important role in the preparation and initiation of spontaneous movements or gait, and in the combination of posture. It is estimated that the decrease in activity of the SMA contributes to the hesitation and FOG at the start of PD patients. FOG is known to appear due to a decrease in structural connectivity between SMA and pedunculopontine nucleus, which pedunculopontine nucleus plays an important role in regulating locomotion. Patients with PD have decreased nigrostriatal dopaminergic neurons, resulting in changes in the morphology of thalamocortical connections, including SMA. In addition, in PD patients, SMA activity is markedly reduced, and structural and functional connections with the mesencephalic locomotor region, which play an important role in postural and motor control, are reduced, resulting in various disorders [26,27].

However, in the previous studies, tDCS was most often applied to the primary motor cortex to improve motor function in PD patients, followed by premotor or prefrontal cortex. Few studies have been applied to SMA, which plays a significant role in initial hesitation, FOG and gait [24,25,28]. Additionally, there are few studies of tDCS combined with visual cueing training.

The first purpose of this study was to investigate the effect of tDCS application on SMA combined with visual cueing training on motor function, balance and gait ability in PD patients. Second, this study was to investigate the difference between the application of tDCS and sham tDCS. Through this, this study was to investigate whether the application of tDCS actually affects the brain and whether it has a placebo effect. Third, this study was to determine whether the tDCS intervention effect persists through follow-up testing. As such, we would like to suggest whether the application of tDCS is useful as an adjuvant therapy to physical therapy by confirming the effects on PD patients.

## 2. Materials and Methods

### 2.1. Participants

The subjects were 30 PD patients from 50 to 75 years old, both genders, and the onset date was more than 3 months. All patients were recruited through the inpatient of the Y rehabilitation hospital.

The inclusion criteria were as follows: (1) independent walking without using walking aid, (2) less than 3 stage on Hoehn and Yahr scale, (3) more than 24 points on Mini Mental State Examination-Korean (MMSE-K) and (4) ON medication state (defined as the patients taking their normal daily medications in the optimally medicated state) [29].

The exclusion criteria were as follows: (1) Severe cognitive or psychological impairment, (2) history of seizure, (3) severe dizziness, (4) device inserted into the heart or brain, (5) orthopedic problems of the lower extremities, (6) impaired vision or hearing, and (7) other tDCS contraindications.

The sample size for this study was calculated using the G* Power program (G power program Version 3.1, Heinrich-Heine-University Dusseldorf, Germany). Based on data from a previous study, the estimated sample size required to obtain a minimum power of 80% at a significant alpha level of 95% was 30 (effect size f 0.28, α err prob 0.05, power (1-β err prob) 0.95) [30]. The potential dropout rate was expected to be 10%, and the total required participants was determined to be 33.

Informed consent was obtained from all patients after sufficient explanation of the procedures. The study was approved by the Ethics Committee of Daegu University (1040621-202101-HR-011).

### 2.2. Experimental Procedure

**Figure 1:**
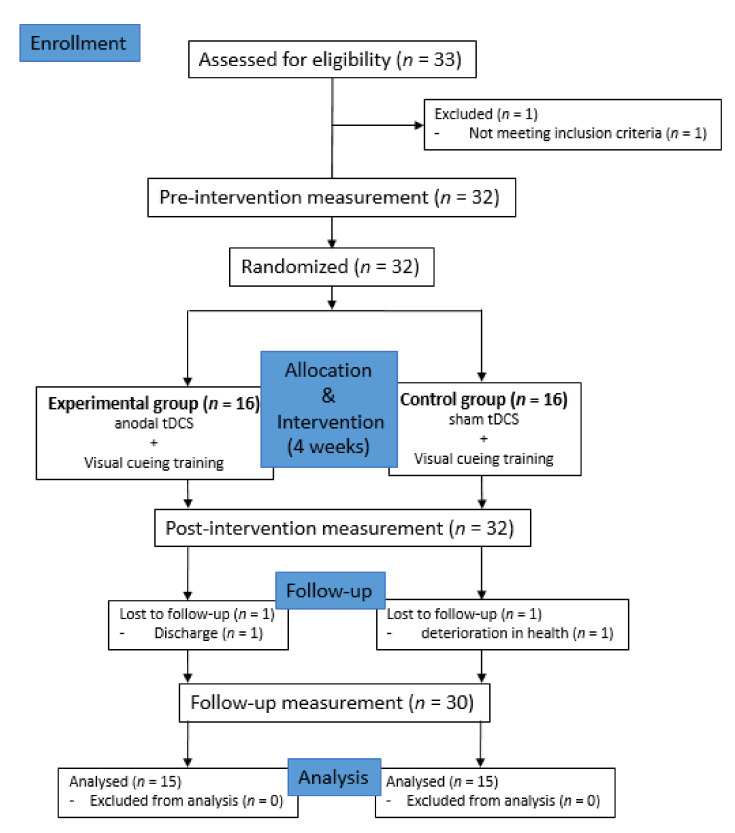
Study flow chart.; Transcranial Direct Current Stimulation (tDCS)

A randomized, single-blind, placebo-controlled study was performed to compare for two groups in PD patients. A computer-based lottery program was used to randomly divide the recruited subjects into two groups. The experimental group (*n* = 15) received real tDCS combined with visual cueing training, and the control group (*n* = 15) received sham tDCS combined with visual cueing training. All subjects received equally 30 min of general physical therapy and 30 min of occupational therapy throughout the day, except for tDCS. All subjects were blinded to group assignment until the study was completed. Figure 1 shows the timeline of tDCS protocol.

#### 2.2.1. Transcranial Direct Current Stimulation (tDCS)

TDCS was applied 20 sessions for 4 weeks when ON state of participants. Using the battery-driven DC-STIMULATOR PLUS (Neuroconn, Ilmenau, Germany), the patient received tDCS at the same time as visual cueing training. The electrode size was 5 × 7 cm, and the pad soaked in saline was used when applying the electrode. The current intensity was 2 mA and applied for 20 min. The anodal tDCS was placed 3 cm in front of the primary motor cortex (Cz) according to the international 10–20 electroencephalography (EEG) system for SMA stimulation, and the cathodal tDCS was placed in the frontal cortex (FP2) of the right orbit (Atsushi et al., 2018). In sham tDCS, the electrode was applied to the same position as the actual tDCS for 20 min, but in order not to stimulate the patient’s brain, it was activated only for the first 30 s, and then the power was turned off [31].

#### 2.2.2. Visual Cueing Training

In general, cueing used for cueing training includes auditory cueing and visual cueing. In this study, visual cueing was used. The first method of visual cueing training was to walk along the shape of the footsteps arranged in a straight line along the walking path. Subject was instructed not to drag their feet, but to lift surely and move to the shape of the next footstep. The second visual cueing training is to walk around two obstacles in an 8-shape rotation for direction change training. The distance between the two obstacles was set to 3 m, and the shape of the footprint was placed on the ground along the walking direction. As with straight walking training, the subject was instructed to lift the foot surely. The visual cueing training performed for this study was used by modifying the method of Pieter et al.’s study [32].

### 2.3. Measurements

#### 2.3.1. Unified Parkinson’s Disease Rating Scale (UPDRS)

UPDRS was used to evaluate the motor function of subjects in this study. UPDRS is the most used measure for clinimetric evaluation of PD patients since it was developed in 1980. By correcting and complementing the shortcomings of the former UPDRS, the Movement Disorders Association has officially introduced and used it by announcing a new version of UPDRS in 2008. UPDRS consists of a total of 65 items in 4 sections. The score of each item is on a 5-point scale from 0 to 4, and the higher the score means the more severe the symptom. Korean-version of the UPDRS part III was used for this study. Previous study showed that UPDRS part III has excellent internal consistency (α = 0.93) [33].

#### 2.3.2. Functional Gait Assessment (FGA)

FGA was used to evaluate balance of subjects in this study. FGA is a 10-item ambulation-based balance test originally developed to evaluate the balance of patients with vestibular disorders, and it is currently used in various diseases for balance evaluation [34]. Berg balance scale, which is commonly used for balance evaluation, has many test items for static balance, while FGA contains many dynamic tasks that require posture adjustment. It is a 4-point scale from 0 to 3, the higher the score, the better the balance. FGA showed high interrater reliability in a study of healthy adults aged 40 to 89 years (intraclass correlation coefficient [ICC] 0.93), and also showed high interrater and test–retest reliability in patients with vestibular disorders (ICC 0.86 and 0.74, respectively) [35]. Korean-version of the FGA was used for this study.

#### 2.3.3. Freezing of Gait Questionnaire (FOG-Q)

FOG-Q was used to evaluate gait ability of subjects in this study. FOG-Q was developed by Giladi et al. and is the only validated measure for assessing FOG severity. This is composed of a total of 6 items, of which 4 are items that evaluate the severity of FOG, and the other two are items that evaluate difficulty in walking. The score of each item is on a 5-point scale from 0 to 4, and the higher the score means the more severe the symptom. Previous study showed that FOG has excellent test-retest reliability and internal consistency (α = 0.94) [36].

#### 2.3.4. Spatio-Temporal Variables of Gait

In order to measure the spatio-temporal variables in the gait process of the subjects, the GAITRite system (CIR Systems Inc. Clifton, NJ, USA), a gait evaluation equipment with proven validity and reliability, was used. GAITRite is a flexible electronic walkway with a width of 61 cm and a length of 366 cm, with 6 sensor pads built in. Each pad has 2304 sensors located in a 48 × 48 grid pattern to detect foot pressure while walking. The load on the foot is collected at a sample rate of 80 Hz per second, and the data is transmitted to a computer by a serial interface cable.

For the evaluation, subjects were instructed to start walking 2 m before the walkway and to stop walking after walking 2 m longer than the end of the walkway. The reason was to exclude the effects of acceleration and deceleration at the beginning and end of walking. In this study, gait velocity, cadence, step time, double support time, and stride length were analyzed among the data obtained through GAITRite. The step time and double support time were evaluated using coefficient of variation (CV%).

A previous study found that GAITRite System has an excellent correlations (ICC 0.95) for spatial measures and excellent correlations (ICC 0.93) for temporal measures [37].

### 2.4. Data Analysis

The normality test of the variable was performed using the Shapiro–Wilk test. Chi-square (χ²) test and independent *t* test were used to find out the homogeneity between groups according to general characteristics. Independent *t* test was performed for comparison between the two groups, and one-way repeated analysis of variance (ANOVA) was used to compare each group before and after intervention and follow-up by measurement period. Two-way repeated ANOVA was performed to verify the interaction between the measurement period and the group, and the intra-group effect according to the measurement period, and the Bonferroni test was used for post hoc analysis. Data were expressed as mean ± standard deviation (SD) and statistical analysis was performed using SPSS version 20.0 (IBM Corporation, Armonk, NY, USA). *p* values less than 0.05 was considered to be statistically significant.

## 3. Results

A total of 33 people was recruited, but three of them were excluded for reasons such as discharge or health deterioration. Thus, a total of 30 subjects participated in this study (experimental group *n* = 15, control group *n* = 15). Table 1 presents the basic characteristics data of the subjects. There were no statistically significant differences in the characteristics of the subjects between the two groups (Table 1).

**Figure 2:**
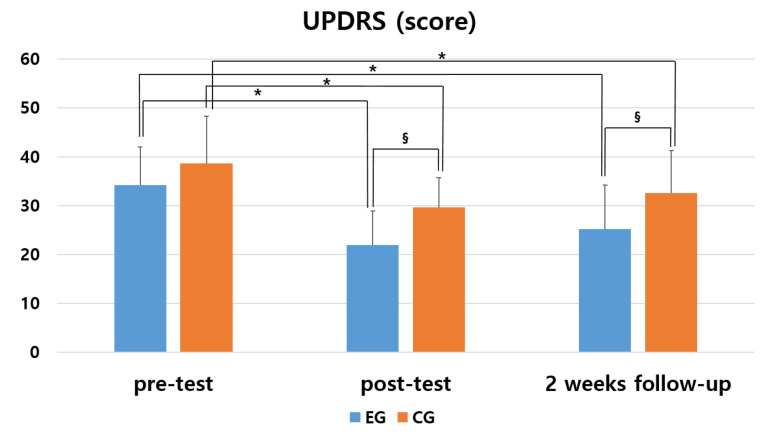
Comparison of the changes of within and between groups in UPDRS. * means a statistical significance within the group as compared to pre (*p* < 0.05). § means a statistical significance by measurement period between groups (*p* < 0.05).

In the Unified Parkinson’s Disease Rating Scale (UPDRS) to evaluate motor function, the experimental group showed a significant difference in post and follow-up test than in pre-test (*p* < 0.05). The control group also showed a significant difference in post and follow-up test than in pre-test (*p* < 0.05). In comparison between the two groups, the experimental group showed a significant difference in post and follow-up test compared to the control group (*p* < 0.05) (Table 2 and Figure 2).

As a result of comparing the amount of change before and after the intervention between the two groups, the experimental group and the control group showed a significant difference between the groups (F = 11.422, *p* = 0.002). There was a significant difference according to the measurement period in the intra-group effect verification (F = 15.820, *p* = 0.000), and the interaction between the measurement period and the group was not significant (F = 0.415, *p* = 0.662). The effect size was 1.247 and 0.885 in the experimental group and the control group, respectively.

**Table 2:** Changes in Unified Parkinson’s Disease Rating Scale (UPDRS) by the period.

Group	Pre	Post	2 Weeks Follow-up	F	*p*
EG ^2,^^‡^	34.20(7.82) ^1^	21.93(6.90) ^†^	25.20(8.99) ^†^	10.884	0.000 *
CG ^3,^^‡^	38.67(9.60)	29.60(6.13) ^†^	32.60(8.70) ^†^	5.482	0.013 *
*t*	−1.397	−3.217	−2.290		
*p*	0.173	0.003 *	0.030 *		

^1^ Mean (± standard deviation). ^2^ Experimental group. ^3^ Control group. * Statistical significance *p* < 0.05. ^†^ *p* < 0.05 as compared to pre (intragroup comparison). ^‡^ effect size greater than 0.70.

**Figure 3:**
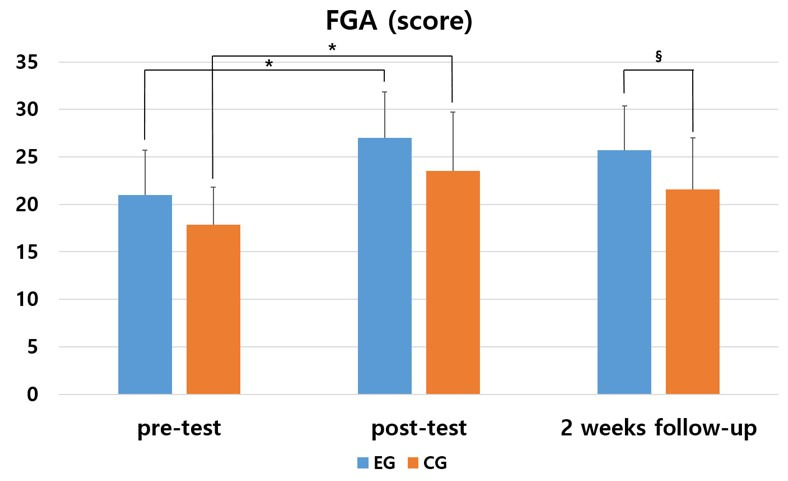
Comparison of the changes of within and between groups in FGA. * means a statistical significance within the group as compared to pre (*p* < 0.05). § means a statistical significance by measurement period between groups (*p* < 0.05).

In the Functional Gait Assessment (FGA) to evaluate balance, the experimental group showed a significant difference in post-test than in pre-test (*p* < 0.05). The control group also showed a significant difference in post-test than in pre-test (*p* < 0.05). In comparison between the two groups, the experimental group showed a significant difference in follow-up test compared to the control group (*p* < 0.05) (Table 3 and Figure 3).

As a result of comparing the amount of change before and after the intervention between the two groups, the experimental group and the control group showed a significant difference between the groups (F = 12.594, *p* = 0.001). There was significant difference according to the measurement period in the intra-group effect verification (F= 10.357, *p* = 0.000), and the interaction between the measurement period and the group was not significant (F = 0.074, *p* = 0.929). The effect size was 0.987 and 0.762 in the experimental group and the control group, respectively.

**Table 3:** Changes in Functional Gait Assessment (FGA) by the period.

Group	Pre	Post	2 Weeks Follow-up	F	*p*
EG ^2,^^‡^	21.00(4.69) ^1^	27.00(4.87) ^†^	25.73(4.67)	6.820	0.004 *
CG ^3,^^‡^	17.87(3.96)	23.53(6.22) ^†^	21.60(5.40)	4.063	0.036 *
*t*	1.977	1.700	2.244		
*p*	0.058	0.100	0.033 *		

^1^ Mean (± standard deviation). ^2^ Experimental group. ^3^ Control group. * Statistical significance *p* < 0.05. ^†^ *p* < 0.05 as compared to pre (intragroup comparison). ^‡^ effect size greater than 0.70.

**Figure 4:**
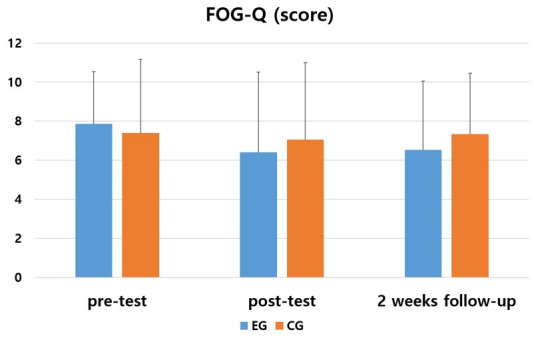
Comparison of the changes of within and between groups in FOG-Q.

In the Freezing of Gait Questionnaire (FOG-Q) to evaluate gait ability, the experimental and the control group showed no significant difference in post and follow-up test than in pre-test (*p* > 0.05). In comparison between the two groups showed no significant difference between the experimental and the control group (*p* > 0.05) (Table 4 and Figure 4).

As a result of comparing the amount of change before and after the intervention between the two groups, there was no significant difference between the experimental group and the control group (F = 0.109, *p* = 0.743). There was no significant difference according to the measurement period in the intra-group effect verification (F = 0.881, *p* = 0.420), and the interaction between the measurement period and the group was not significant (F = 0.478, *p* = 0.623).

**Table 4:** Changes in Freezing of Gait Questionnaire (FOG-Q) by the period.

Group	Pre	Post	2 Weeks Follow-up	F	*p*
EG ^2^	7.866(2.66) ^1^	6.40(4.11)	6.53(3.52)	0.914	0.413
CG ^3^	7.40(3.77)	7.06(3.95)	7.33(3.13)	0.106	0.850
*t*	0.391	−0.452	−0.657		
*p*	0.699	0.655	0.516		

^1^ Mean (± standard deviation). ^2^ Experimental group. ^3^ Control group.

**Figure 5:**
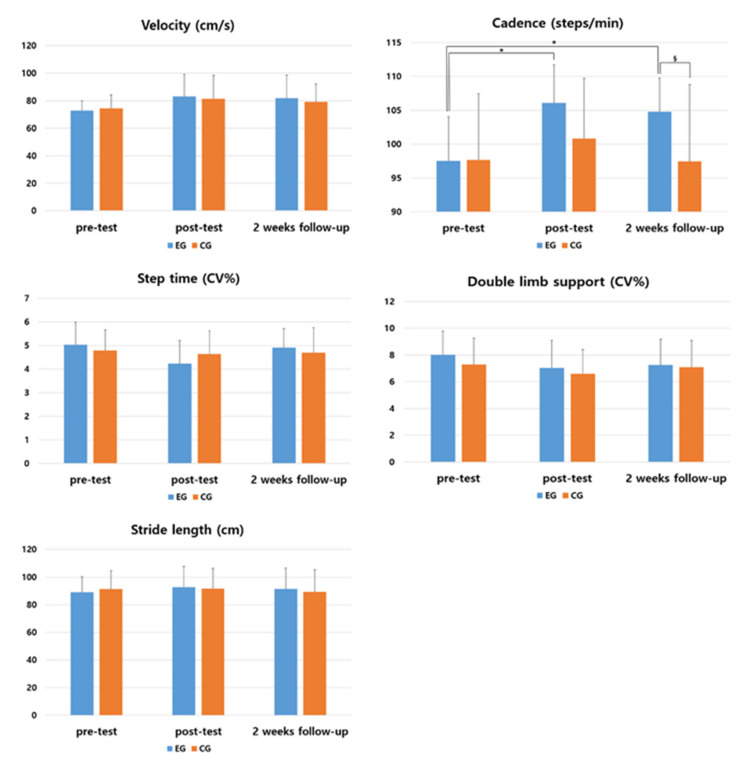
Comparison of the changes of within and between groups in gait variables. * means a statistical significance within the group as compared to pre (*p* < 0.05). § means a statistical significance by measurement period between groups (*p* < 0.05).

In the evaluation of gait velocity, the experimental and the control group showed no significant difference in post and follow-up test than in pre-test (*p* > 0.05). In comparison between the two groups showed no significant difference between the experimental and the control group (*p* > 0.05) (Table 5 and Figure 5).

As a result of comparing the amount of change before and after the intervention between the two groups, there was no significant difference between the experimental group and the control group (F = 0.038, *p* = 0.846). There was significant difference according to the measurement period in the intra-group effect verification (F = 4.61, *p* = 0.054), and the interaction between the measurement period and the group was not significant (F = 2.31, *p* = 0.779).

**Table 5:** Changes in gait velocity by the period.

Group	Pre	Post	2 Weeks Follow-up	F	*p*
EG ^2^	72.80(6.90) ^1^	83.00(16.17)	81.79(16.87)	3.337	0.050
CG ^3^	74.40(9.75)	81.53(16.75)	79.26(12.86)	1.489	0.243
*t*	−0.518	0.244	0.414		
*p*	0.608	0.809	0.682		

^1^ Mean (± standard deviation). ^2^ Experimental group. ^3^ Control group.

In the evaluation of cadence, the experimental group showed a significant difference at the post and follow-up test than the pre-test (*p* < 0.05). In comparison between the two groups, the experimental group showed a significant difference in follow-up test compared to the control group (*p* < 0.05) (Table 6 and Figure 5).

As a result of comparing the amount of change before and after the intervention between the two groups, the experimental group and the control group showed a significant difference between the groups (F = 5.944, *p* = 0.021). There was a significant difference according to the measurement period in the intra-group effect verification (F = 3.799, *p* = 0.028), and the interaction between the measurement period and the group was not significant (F = 1.635, *p* = 0.204). The effect size was 1.316 in the experimental group.

**Table 6:** Changes in cadence by the period.

Group	Pre	Post	2 Weeks Follow-up	F	*p*
EG ^2,^^‡^	97.53(6.52) ^1^	106.07(5.65) ^†^	104.80(4.96) ^†^	12.114	0.000 *
CG ^3^	97.67(9.79)	100.80(8.95)	97.47(11.33)	0.476	0.626
*t*	−0.044	1.927	2.296		
*p*	0.965	0.064	0.029 *		

^1^ Mean (± standard deviation). ^2^ Experimental group. ^3^ Control group. * Statistical significance *p* < 0.05. ^†^ *p* < 0.05 as compared to pre (intragroup comparison). ^‡^ effect size greater than 0.70.

In the evaluation of step time, the experimental and the control group showed no significant difference in post and follow-up test than in pre-test (*p* > 0.05). In comparison between the two groups showed no significant difference between the experimental and the control group (*p* > 0.05) (Table 7 and Figure 5).

As a result of comparing the amount of change before and after the intervention between the two groups, there was no significant difference between the experimental group and the control group (F = 0.00, *p* = 0.934). There was no significant difference according to the measurement period in the intra-group effect verification (F = 1.912, *p* = 0.160), and the interaction between the measurement period and the group was not significant (F = 1.064, *p* = 0.349).

**Table 7:** Changes in step time by the period.

Group	Pre	Post	2 Weeks Follow-up	F	*p*
EG ^2^	5.03(0.95) ^1^	4.23(0.98)	4.90(0.81)	2.590	0.097
CG ^3^	4.79(0.87)	4.64(0.97)	4.69(1.06)	0.101	0.894
*t*	0.721	−1.145	0.620		
*p*	0.477	0.262	0.540		

^1^ Mean (± standard deviation). ^2^ Experimental group. ^3^ Control group.

In the evaluation of double support time, the experimental and the control group showed no significant difference in post and follow-up test than in pre-test (*p* > 0.05). In comparison between the two groups showed no significant difference between the experimental and the control group (*p* > 0.05) (Table 8 and Figure 5).

As a result of comparing the amount of change before and after the intervention between the two groups, there was no significant difference between the experimental group and the control group (F = 1.340, *p* = 0.257). There was no significant difference according to the measurement period in the intra-group effect verification (F= 1.330, *p* = 0.273), and the interaction between the measurement period and the group was not significant (F = 0.145, *p* = 0.864).

**Table 8:** Changes in double support time by the period.

Group	Pre	Post	2 Weeks Follow-up	F	*p*
EG ^2^	8.01(1.76) ^1^	7.02(2.07)	7.25(1.93)	1.112	0.343
CG ^3^	7.29(1.98)	6.60(1.81)	7.08(2.00)	0.427	0.524
*t*	1.053	0.592	0.232		
*p*	0.301	0.558	0.818		

^1^ Mean (± standard deviation). ^2^ Experimental group. ^3^ Control group.

In the evaluation of stride length, the experimental and the control group showed no significant difference in post and follow-up test than in pre-test (*p* > 0.05). In comparison between the two groups showed no significant difference between the experimental and the control group (*p* > 0.05) (Table 9 and Figure 5).

As a result of comparing the amount of change before and after the intervention between the two groups, there was no significant difference between the experimental group and the control group (F = 0.005, *p* = 0.946). There was no significant difference according to the measurement period in the intra-group effect verification (F = 1.041, *p* = 0.356), and the interaction between the measurement period and the group was not significant (F = 1.148, *p* = 0.322).

**Table 9:** Changes in stride length time by the period.

Group	Pre	Post	2 Weeks Follow-up	F	*p*
EG ^2^	89.00(11.07) ^1^	92.53(15.02)	91.47(14.89)	1.472	0.246
CG ^3^	91.13(13.53)	91.53(14.64)	89.33(15.82)	0.678	0.505
*t*	−0.473	0.185	0.380		
*p*	0.640	0.855	0.707		

^1^ Mean (± standard deviation). ^2^ Experimental group. ^3^ Control group.

## 4. Discussion

In PD patients, interventions to improve movement, balance, and gait are important to reduce the risk of falls and increase independence in daily activities. Therefore, the aim of this study was to investigate the effect of tDCS combined with visual cueing training on motor function, balance, and gait function in PD patients. No adverse effects of tDCS were observed in this study.

The experimental group showed significant improvement compared to the control group in the test using UPDRS to determine the change in motor function. Recent studies have shown that epidural stimulation through non-invasive brain stimulation can improve motor function in PD patients [38]. In the study of applying rTMS to SMA of PD patients, it was reported that fine motor function was improved and the threshold for excitability in other motor areas was decreased [39]. According to Valentino et al., there was no significant effect on the UPDRS score after 5 tDCS sessions, but when assessing the delayed effect after stimulation, it was confirmed that there was a significant effect compared to the control group [25]. In this study, as well as the evaluation of delayed effect through follow-up studies, there was a significant effect in UPDRS score before and after intervention compared to the control group, which is thought to be due to the difference between the total treatment session of the study by Valentino et al.

There are two pathophysiological mechanisms for cortical stimulation to improve PD symptoms. The first mechanism is an increase in neurotransmitters. Cortical stimulation is linked to basal ganglia function and can induce changes throughout the cortical-subcortical network. These remote effects are related to the release of specific neurotransmitters [22]. The second mechanism is normalization of cortical function. According to a previous study, it was confirmed that the reduced intracortical inhibition and corticospinal output in basal ganglia connected areas such as the SMA in the functional magnetic resonance imaging of PD patients [40]. tDCS can correct and improve the networking ability of neurons in PD patients with basal ganglia dysfunction by effectively reaching the cortico-subthalamic projection, which is involved in motor coordination by penetrating the cortex of the brain. For this reason, it is considered that there was a significant improvement in the motor function of the experimental group in this study.

The experimental group showed significant improvement compared to the control group in the test using FGA to determine the change in balance. Previous studies reported that anodal tDCS had a significant effect on balance and functional mobility compared to sham tDCS. The study of Lattari et al. used dorsolateral prefrontal cortex (DLPFC) as the stimulation location, which is important in that DLPFC is a major brain area that compensates for SMA activity disorders [41]. In a study by Andrade et al. in stroke patients, it was reported that the three groups that received tDCS stimulation showed significant improvement in gait and balance compared to the sham tDCS group [42].

Conversely, the results of this study showed that the control group also showed improvement in motor function and balance, which needs to be noted for the effect of visual cueing training. According to a case study using EEG, it was reported that visual cueing during walking increased the flow of the occipito-parietal-motor network in PD patients [43]. In addition, visual cueing is believed to have a positive effect on improving the timing of movement and the amplitude of gait more appropriately and spatio-temporal stabilization, rather than increasing attention itself [44]. These mechanisms suggest that there would have been improvements in UPDRS and FGA scores in control group with visual cueing training.

FOG-Q and GAITRite system were used to investigate changes in the gait ability of PD patients in this study. The experimental group showed significant improvement only in cadence among several gait-related variables. In the study of Chiahao et al., similar to this study, anodal tDCS was applied to SMA of PD patients, but reported that there was no significant effect on FOG [28]. There was a difference between the previous study and this study in the application of tDCS, tDCS was applied simultaneously with physical training for 20 min using 2 mA in this study, but Chiahao et al. applied anodal tDCS for 10 min before physical training using 1 mA. However, the findings that there was no significant effect on the freezing phenomenon in PD patients are consistent between the two studies.

This study showed that tDCS had a significant effect on cadence among gait variables. A common finding that appeared when SMA was stimulated in previous studies was a change in temporal features of the task. Applying anodal tDCS to healthy adults showed a decrease in reaction times, and a study in PD patients showed a decrease in the time required to complete a motor tasks, such as turning or walking [7]. Benninger et al. reported a significant improvement in gait and bradykinesia compared to sham tDCS after alternately applying anodal tDCS to the pre- and motor cortex and the prefrontal cortex in PD patients [24]. Previous studies that analyzed the effect of tDCS combined with cueing training showed a significant improvement in several measurements of gait-related outcome compared to sham tDCS when anodal tDCS was applied to primary motor cortex [45]. Another study reported that the group that combined cueing training and tDCS had a faster effect than the group that only did cueing training, and that the effect lasted longer until 1 month follow-up [29].

In addition, previous studies have found a tendency to have a positive correlation between the increase in cortical excitability and improvement in motor function [20,29]. Therefore, in this study applying anodal tDCS, it is believed that the increase in cortical excitability had a positive effect on the improvement of gait function. However, it did not satisfy the hypothesis of this study that the experimental group would have a significant difference compared to the control group in all the gait variables. In general, the stimulation intensity of tDCS is 1 mA and 2 mA, and there are two main reasons for applying 2 mA in this study. First, it is known that the greater the stimulation intensity of tDCS, the greater the effect. Boggio et al. investigated the effect on accuracy in a working memory after stimulation using 1 mA and 2 mA, showing a much greater effect at 2 mA, and the effect sizes of the two interventions were large and moderate, respectively. Study related to motor learning also showed that high intensity was effective in improving skill acquisition compared to low intensity. This is considered to be because the higher the current intensity, the higher the activity-dependent modification in synaptic efficacy, thereby improving motor learning and motor performance. Second, it is due to its association with dopamine drug intake. According to previous studies, patients with OFF-medication showed improved performance even at 1 mA, but when applied during the ON state, a negative effect on gait function was found [46,47]. Therefore, 2 mA intensity was used for all studies conducted in the ON state of drug cycle, and 2 mA intensity was selected since this study targeted ON-phase patients.

According to a systematic review, it was mentioned that studies applying tDCS showed a consistent trend in that tDCS showed a positive effect on PD patients, although large heterogeneity was found in stimulation parameters and study design [48]. In another study, it was suggested that applying tDCS during motor learning can achieve an effect similar to that of long-term potentiation (LTP) and prolong the performance improvement [49]. As a result of this study, all variables that had significant differences in the comparison between groups showed a significant effect, especially in follow-up. Therefore, it can be seen that it is consistent with the results of previous studies that tDCS can prolong the effect.

However, several previous studies and the results of this study show that combining tDCS with other physical training has a positive effect on motor or gait function, but evidence supporting the effect of tDCS alone is still insufficient. Kaski et al. demonstrated that applying anodal tDCS with physical training to PD patients is effective in improving gait function compared to tDCS or physical training alone [30]. Some studies have concluded that tDCS is beneficial in combination with other training because it can increase motor learning and improve performance of motor tasks [50]. Therefore, this study suggests that tDCS is useful as an adjuvant intervention for rehabilitation training in PD patients, rather than applying it alone.

There are several limitations to this study. Since the electrode size used in this study was 5 × 7 cm, it is possible that tDCS stimulation was not limited to SMA but also affected other motor cortex. In addition, since neuroimaging studies were not included, it was not possible to accurately evaluate the activity in the brain. In addition, in PD patients with fixed gait patterns, the duration of intervention was not long enough to discover the change in gait.

Despite these limitations, the results of this study are meaningful to provide the first evidence that the application of tDCS to SMA has a positive effect on the improvement of motor function, balance and gait ability in PD patients. It could also encourage additional tDCS studies on gait function in PD patients. As yet, tDCS is not widely used as a rehabilitation therapy for PD patients in clinical practice. However, the study using more subjects and various stimulus parameters will be able to confirm the more definite effect than the results of this study, which will be able to find more powerful clinical effects. Future studies will need to evaluate various stimulation parameters such as intensity of tDCS, stimulation location, and number of stimulation sessions.

## 5. Conclusions

This study was conducted to investigate the effect of tDCS applied to SMA in PD patients on motor function, balance, and walking ability. The experimental group that received tDCS combined with visual cueing training showed significant differences in UPDRS for motor function evaluation, FGA for balance evaluation and cadence on the GAITRite system test for gait evaluation after 4 weeks of intervention compared to the control group that received sham tDCS combined with visual cueing training. In particular, it was confirmed that there was a long-term effect through follow-up in all variables showing significant differences. Therefore, based on the results of this study, it is suggested that tDCS combined with physical training can have more positive effects on the motor function, balance and walking ability of PD patients than applying physical training alone. In addition, it is recommended to be widely used in clinical sites as an intervention method for PD patients who need long-term rehabilitation because the duration of the therapeutic effect is extended.

## Figures and Tables

**Table 1:** General characteristics of the subjects.

Variable	EG (*n* = 15) ^3^	CG (*n* = 15) ^4^	*t/x* ^2^	*p*
Gender (male/female)	6/9	8/7	0.714	0.481
Height (cm)	167.33(6.29) ^1^	165.00(7.34)	0.935	0.358
Weight (kg)	63.93(6.41)	61.60(5.46)	1.073	0.292
Education received (years)	12.07(3.71)	13.47(1.77)	−1.319	0.198
Age (years)	70.00(3.76)	71.33(3.27)	−1.037	0.309
Duration (months)	6.27(1.03)	7.00(1.41)	−1.622	0.116
Hoehn and Yahr (stage)	2.47(0.52)	2.80(0.41)	−1.950	0.061
MMSE-K (score) ^2^	26.33(1.35)	26.87(1.51)	−1.023	0.315

^1^ Mean (± standard deviation). ^2^ Mini Mental State Examination—Korean. ^3^ Experimental group. ^4^ Control group.

## Data Availability

The data presented in this study are available on reasonable request from the corresponding author.
